# Glycosylated Ang-(1-7) MasR Agonist Peptide Poly Lactic-co-Glycolic Acid (PLGA) Nanoparticles and Microparticles in Cognitive Impairment: Design, Particle Preparation, Physicochemical Characterization, and In Vitro Release

**DOI:** 10.3390/pharmaceutics14030587

**Published:** 2022-03-08

**Authors:** David Encinas-Basurto, John P. Konhilas, Robin Polt, Meredith Hay, Heidi M. Mansour

**Affiliations:** 1 Skaggs Pharmaceutical Sciences Center, College of Pharmacy, The University of Arizona, Tucson, AZ 85721, USA; dencinas@pharmacy.arizona.edu; 2 Department of Physiology and Sarver Heart Center, The University of Arizona, Tucson, AZ 85721, USA; konhilas@arizona.edu; 3 Department of Chemistry & Biochemistry, The University of Arizona, Tucson, AZ 85721, USA; polt@email.arizona.edu; 4 BIO5 Institute, The University of Arizona, Tucson, AZ 85721, USA; 5 Department of Physiology and Evelyn F. McKnight, Brain Institute, The University of Arizona, Tucson, AZ 85721, USA; mhay@arizona.edu; 6 Division of Translational and Regenerative Medicine, Department of Medicine, The University of Arizona College of Medicine, Tucson, AZ 85721, USA; 7 Center for Translational Science, Florida International University, Port St. Lucie, FL 34987, USA

**Keywords:** PNA5 glycopeptide, mas receptor, angiotensin, PLGA diblock copolymer, ester and acid-end capped, double emulsion solvent evaporation, biocompatible, biodegradable, cardiovascular, neurodegenerative diseases

## Abstract

Heart failure (HF) causes decreased brain perfusion in older adults, and increased brain and systemic inflammation increases the risk of cognitive impairment and Alzheimer’s disease (AD). Glycosylated Ang-(1-7) MasR agonists (PNA5) has shown improved bioavailability, stability, and brain penetration compared to Ang-(1-7) native peptide. Despite promising results and numerous potential applications, clinical applications of PNA5 glycopeptide are limited by its short half-life, and frequent injections are required to ensure adequate treatment for cognitive impairment. Therefore, sustained-release injectable formulations of PNA5 glycopeptide are needed to improve its bioavailability, protect the peptide from degradation, and provide sustained drug release over a prolonged time to reduce injection administration frequency. Two types of poly(D,L-lactic-co-glycolic acid) (PLGA) were used in the synthesis to produce nanoparticles (≈0.769–0.35 µm) and microparticles (≈3.7–2.4 µm) loaded with PNA5 (ester and acid-end capped). Comprehensive physicochemical characterization including scanning electron microscopy, thermal analysis, molecular fingerprinting spectroscopy, particle sizing, drug loading, encapsulation efficiency, and in vitro drug release were conducted. The data shows that despite the differences in the size of the particles, sustained release of PNA5 was successfully achieved using PLGA R503H polymer with high drug loading (% DL) and high encapsulation efficiency (% EE) of >8% and >40%, respectively. While using the ester-end PLGA, NPs showed poor sustained release as after 72 h, nearly 100% of the peptide was released. Also, lower % EE and % DL values were observed (10.8 and 3.4, respectively). This is the first systematic and comprehensive study to report on the successful design, particle synthesis, physicochemical characterization, and in vitro glycopeptide drug release of PNA5 in PLGA nanoparticles and microparticles.

## 1. Introduction

Cases of dementia and heart failure (HF) both represent growing social, healthcare, and economic issues. It is estimated that more than 35 million people worldwide had dementia in 2010, with the number expected to double every 20 years [1,2]. HF causes decreased brain perfusion in older adults, and increased brain and systemic inflammation are known to increase the risk of cognitive impairment and Alzheimer’s disease (AD) [3,4]. Decreased brain blood flow, increased reactive oxygen species (ROS) production, and proinflammatory mechanisms all appear to accelerate neurodegenerative disease progression, as seen in vascular contributions to cognitive impairment and dementia (VCID), AD, and related dementias [5] and, it is still an unmet condition that lacks effective treatment.

The renin-angiotensin system (RAS) is of importance in both cognitive function and HF. There is evidence that activating the ACE-AngII-AT1R pathway (in the RAS system) involves balancing ROS production and nitric oxide (NO) production in the brain. Xu, Sriramula [6] showed that the synthesis of Ang(1-7) by activating the ACE2 pathway correlated to decreased ROS production and increased nitric oxide synthase in the brain. Also, the G-protein-coupled receptor Mas (MasR) is activated by Ang(1-7) coupling. There is evidence that MasR is essential for standard object recognition processing, and the lack of the receptor impairs object recognition [7]. Our group hypothesized that Ang-(1-7) production and increased MasR activation might be beneficial for memory and cognitive function. In previous work, Hay, Vanderah [3] demonstrated that a systemic dose of Ang-(1-7) attenuated and even restored HF-induced cognitive impairment in mice, correlated to neuroprotective biomarkers.

However, it is known that low bioavailability and metabolic liability are observed after peptide or protein administration. High molecular weight, low lipophilicity, and charged functional groups in peptides affect their correct absorption. These features are the reason why most orally administered peptides have a low bioavailability (2%) and short half-life (≈30 min) [8]. Although subcutaneous or intravenous dosing can help with absorption peptide opsonization, conformational changes, subunit protein dissociation, and systemic proteases remain a problem for biotherapeutics appropriate effect [8,9]. Hay, Polt [5] developed a novel Ang-(1-7) glycoside with MasR agonist activity and enhanced pharmacokinetics/pharmacodynamics properties. The Mas agonist Ang-1-6-O-Ser-Glc-NH_2_ (PNA5) glycopeptides were rationally designed to improve bioavailability, stability, and brain uptake; PNA5 glycopeptide may be regarded as a new class of peptide drugs [10]. Previous in vitro work has shown that t_1/2_ of PNA5 in serum samples is 1.06 ± 0.2 h. In vivo, after intravenous injection, bioavailability was improved compared to its native Ang-(1-7), but after 30 min neither PNA5 in serum nor CSF could be detected. Those observations were the motivation behind the encapsulation of PNA5 using FDA-approved polymer PLGA to get a slow and long sustained release to enhance its effects in the treatment and prevention of cognitive impairment. 

Despite promising results and numerous potential PNA5 glycopeptide applications, limitations imposed by their short half-life required frequent injections to ensure adequate VCID treatment. Therefore, the proper formulation is needed for patients with this condition to achieve a long-term and consistent therapeutic effect [11,12,13,14]. Microparticles (MPs) and nanoparticles (NPs) have several advantages over the traditional application of the pure active pharmaceutical ingredient (API), such as increased drug bioavailability (and thus the possibility of reducing drug dosage and resultant side effects) [15]. Particle size has been reported to impact circulation time, cellular uptake, and release behavior. Apart from the significant physical differences between micro and nanoparticles, microparticles are thought to be able to entrap higher concentrations of medicines [16]. The FDA-approved diblock copolymer, poly(d,l-lactic-co-glycolic acid) (PLGA), is biocompatible and biodegradable [17] and has been extensively used for peptide/protein delivery and sustained-release formulations, including several successful pharmaceutical marketed sustained-release injectable products [18,19,20,21]. Depending on the drug type, it is feasible to alter the overall physical properties of the polymer-drug matrix by adjusting important parameters such as polymer molecular weight, lactide-to-glycolide ratio, and drug concentration to achieve a desired dosage and release interval; also, end-capped functional groups are a key component of PLGA [22]. These, in turn, affect peptide drug release from PLGA diblock copolymer and in vivo peptide drug pharmacokinetics [23].

In this comprehensive and systematic study, we successfully prepared PLGA NPs/MPs through a double emulsion solvent evaporation method to allow the successful and efficient encapsulation of PNA5 glycopeptide. Two types of poly(D,L-lactic-co-glycolic acid) (PLGA) were used in the synthesis to produce nanoparticles and microparticles loaded with PNA5 (ester and acid-end capped). Comprehensive physicochemical characterization including scanning electron microscopy, thermal analysis, molecular fingerprinting spectroscopy, particle sizing, drug loading, encapsulation efficiency, and in vitro drug release were conducted. To the authors’ knowledge, this is the first systematic and comprehensive study to report on the successful design, particle synthesis, physicochemical characterization, and in vitro glycopeptide drug release of PNA5 PLGA nanoparticles and microparticles.

## 2. Materials and Methods

### 2.1. Materials

Various types of PLGA (50:50) diblock copolymers were purchased from Evonik (Essen, Germany) including 503H and 503 (≈31 kDa, acid and ester end-capped, respectively). Polyvinyl Alcohol (PVA) (molecular weight 89,000-98,000 g/mol) was purchased from Sigma-Aldrich Chemical Co. (St. Louis, MO, USA). Dichloromethane and acetonitrile (HPLC grade) were obtained from Sigma-Aldrich (St. Louis, MO, USA). Glycosylated peptide (PNA5) was synthesized by the PolyPeptide Group, Torrence, CA via solid-phase synthesis.

### 2.2. Methods

#### 2.2.1. Preparation of PNA5 PLGA NPs/MPs Using Double Emulsion Solvent Evaporation 

PLGA NPs and MPs loaded with PNA5 peptides were prepared by the double-emulsification solvent evaporation method (W_1_/O/W_2_), with two esters and acid terminated PLGA 50:50 as a polymeric system (R503 and R503H, respectively), conditions and variables applied for the preparation of PNA5- micro/nanoparticles are shown in Table 1. Briefly, PLGA was dissolved in dichloromethane (DCM), forming the oil phase (O), then water phase in the first emulsion (W_1_) was slowly added to the oil phase and sonicated at 50% amplitude for 180 s (Sonics Vibra-Cell vcx-500, Sonics & Materials, Inc., Newtown, CT, USA) to get the first emulsion (E_1_), then E_1_ was added to 2% w/v PVA solution (W_2_) to form a second emulsion (E_2_), and an E_2_ was formed, either with homogenization (4500 rpm, Silverson L5M, Silverson Machines, Inc., East Longmeadow, MA, USA) or sonication (50% amplitude for 60 s) for MP and NPs, respectively. Furthermore, for PLGA MPs synthesis, E_2_ was added to the W_3_ phase to solidify the microspheres. After 3 h of stirring (oil phase solvent evaporation), MP and NPs were transferred to centrifuge tubes, washed three times (4000 rpm, 10 min, 10,000 rpm, 20 min, respectively) with distilled water. Before the freeze-drying process, particles were resuspended in a trehalose solution in a 1:2 weight ratio (PLGA:Trehalose).

#### 2.2.2. Characterization of the Prepared PNA5-Loaded PLGA MP/NPs 

##### Particle Size, Size Distribution, and ζ Potential Measurements

The Z-average size and polydispersity index (PDI) of particles were measured by dynamic light scattering, using a Zetasizer (Nano ZS, Malvern Ltd., Malvern, UK). The zeta-potential, ζ, was measured by electrophoretic mobility using the same instrument. For that purpose, MP/NPs were diluted to 0.1× PBS, pH 7.4.

##### Scanning Electron Microscopy (SEM) Measurements

SEM was used to examine particle size and shape (FEI Inspect S microscope, FEI, Brno, Czech Republic). Double-side adhesive carbon tape was used to fix the samples to aluminum stubs (TedPella, Inc., Redding, CA, USA). An Anatech Hummer 6.2 sputtering system was used for sample gold-coating at 15 AC milliAmperes with about 7 kV of voltage for 90 s, getting 7 nm gold-thin film on the powder using a 9–12.5 mm working distance.

##### Encapsulation Efficiency (EE) and Drug loading (DL)

Peptide DL was determined by measuring the peptide content of dissolved particles by reversed-phase HPLC. For that purpose, 1 mg of dry particles was dissolved in 1 mL DMSO, and the solution was agitated at 37 °C for 60 min. Next, 10 mL MQ water was added, and the mixture was agitated at 37 °C for 60 min, to allow peptide extraction. A volume of 20 µL of the solution was filtered and injected into an HPLC system equipped with a C18 column. % EE was measured by HPLC injection of the first centrifuge supernatant of the NP/MPs. The drug-loading content (DLC %) and the drug entrapment efficiency (EE %) were calculated as shown in Equations (1) and (2), respectively:

% DL = (Weight of Drug in NPs/Weight of NPs) × 100(1)

% EE = (Actual PNA5/Theoretical PNA5 Content) × 100(2)

##### Chromatographic Equipment and Conditions

The analysis of PNA5 was performed by a reverse phase HPLC assay, using Phenomenex Prodigy 5 um ODS (3) 250 × 4.6 mm in an LC-2010HT next-generation HPLC (SHIMADZU, Tokyo, Japan). Mobile phase conditions were a gradient of acetonitrile (CH_3_CN) in water (H_2_O) with 0.1% trifluoroacetic acid (CF_3_COOH) at a 1 mL/min flow rate. The start condition used was solvent A: solvent B in a ratio of 95:5; after 24 min, the ratio was 5:95. Solvent A composed 95% ACN and % 5 of the aqueous phase; solvent B was 20% ACN and 80% aqueous phase; the total run time was 24 min. Volume injection was 20 µL and the retention time for PNA5 was ~6 min. 

#### 2.2.3. Attenuated Total Reflectance (ATR) Fourier Transform Infrared Spectroscopy (FTIR)

A Nicolet Avatar 360 FTIR spectrometer (Varian Inc., Palo Alto, CA, USA) equipped with a DTGS detector and an attenuated total reflectance (ATR) accessory was used to record the spectra of raw PNA5 glycopeptide, PLGA diblock copolymer, and PNA5-loaded PLGA particles. Each spectrum was obtained across a wavenumber range of 4000–400 cm^−1^ for 32 scans at a spectral resolution of 2 cm^−1^. Under the same experimental settings, a background spectrum was created. Data analysis was collected using the EZ OMNIC 9.1 software.

#### 2.2.4. Differential Scanning Calorimeter (DSC)

Raw PNA5-PLGA formulations were investigated using thermal analysis and phase transition measurements. Thermograms were collected using a TA Q1000 differential scanning calorimeter (DSC) with T-Zero^®^ technology, an automated computer-controlled RSC-90 cooling accessory, and an autosampler (TA Instruments, New Castle, DE, USA). In a hermetic anodized aluminum DSC pan, a mass of a 3–5 mg sample was accurately weighed. Using the T-Zero^®^ hermetic press, the T-Zero^®^ DSC pans were hermetically sealed (TA Instruments). As a baseline, an empty hermetically sealed aluminum pan was employed. From 0.00 °C to 300 °C, DSC measurements were made at a rate of 5.00 °C/minute.

#### 2.2.5. In Vitro Release of Peptide PLGA Nanoparticles and Microparticles

After resuspending 10 mg of freeze-dried MP/NPs in 10 mL of PBS buffer, the resulting particle suspension was incubated at 37 °C with magnetic stirring (200 rpm, *n* = 3). At certain time intervals, aliquots were taken, samples were centrifuged, and the supernatants were collected, followed by pellet resuspension in fresh PBS. The peptide concentration in the supernatant was determined with HPLC at 280 nm by comparing the concentration to a previously constructed standard calibration curve.

PNA5 in vitro drug release was fitted using four different kinetic models: zero-order, first-order, Higuchi, and Korsmeyer-Peppas.

Zero-order kinetics were fit to Equation (3):

(3) $$Q_{t}-Q_{0}=K_{0}t$$

where *Q_t_* is the amount of drug released after time *t*, *Q*_0_ is the initial amount of drug in solution and *K*_0_ is the zero-order rate constant. 

First-order kinetics were fit to Equation (4):

(4) $$\ln Q_{t}=\ln Q_{0}-K_{1}t$$

where *Q_t_* is the amount of drug released after time *t*, *Q*_0_ is the initial amount of drug in solution and *K*_1_ is the first-order rate constant. 

Higuchi kinetics were fit to Equation (5):

(5) $$Q_{t}=K_{H}\times t^{1/2}$$

*K_H_* is the Higuchi dissolving constant, and *Q_t_* is the amount of drug released after time *t*.

Finally, release kinetics were fit to the Korsmeyer-Peppas model, Equation (6):

(6) $$Q_{t}=Kt^{n}$$

where *Q_t_* is the amount of drug released after time *t*, *K* is the rate constant and *n* is the diffusion exponent for drug release. 

## 3. Results 

### 3.1. Characterization of the Prepared PNA5-Loaded PLGA MP/NPs 

The influence of PLGA type (503H and 503) on the properties of PNA5-loaded particles was investigated in this study and for that, we generated two types of particles (NPs and MPs). As shown in Table 2, the diameter of the PLGA-based NPs, as determined by dynamic light scattering, was 0.76 µm and 0.35 µm for R503 and R503H, respectively. While the size of MPs was larger for R503 than 503H MP (3.7 µm versus 2.4 µm). Particles from both polymer types (R503 and R503H) and sizes (NP/MP) showed a negative surface charge higher for acid terminated PLGA of −30.4 ± 1.9 mV and 32.2 ± 2.5 mV for NP and MP, respectively. On the other hand, 503 polymers showed a ζpotential of −25 ± 1.5 and −24 ± 2.3 for NP and MP, respectively.

To optimize the maximum amount of PNA5 encapsulated and to have a sustained release, we evaluated the two PLGA polymers (R503 and R503H) as NP and MPs. For NPs synthesis, an encapsulation efficiency (EE) of 10.8 and 42% was observed for R503 and R503H, respectively. For MPs, EE’s of 13% and 55% were observed for 503 and 503H respectively. Regarding % drug loading (DL), MPs formulations yield a higher value than NPs. For example, when comparing the DL of 503H MPs to 503H NPs, the 503H MP showed a DL of 9.3% while NPs had a DL of 8.3%, with this trend being present in PLGA 503 particles.

**Figure 1:**
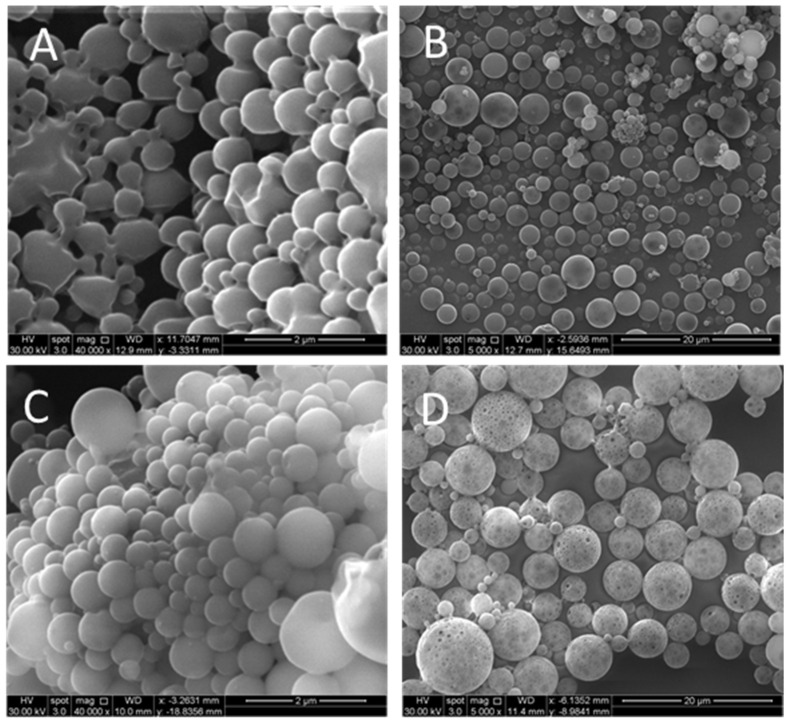
Scanning electron microscopy (SEM) images of microparticles (MPs) and nanoparticles (NPs) synthesized by double emulsion solvent evaporation for PLGA 503H (panels **A**,**B**) and PLGA 503 (panels **C**,**D**) diblock copolymers.

The PNA5-loaded PLGA particles SEM micrographs are shown in Figure 1. Figure 1A,C are PLGA NPs using R503 and R503H. Figure 1B,D are for MPs of 503 and 503H, respectively. Since the optimized formula was uniform (according to low PDI), we chose a single image as a representation for each formulation. The results showed that PNA5-loaded PLGA NPs with a relatively smooth surface were spherical and had a homogenous distribution consistent with the abovementioned results showing low PDI values in all formulations.

### 3.2. Attenuated Total Reflectance (ATR) Fourier Transform Infrared Spectroscopy (FTIR)

**Figure 2:**
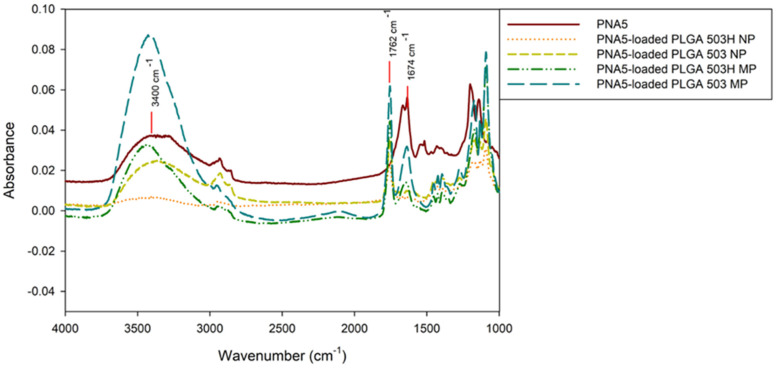
ATR FT-IR molecular fingerprinting spectra of Raw PNA5 glycopeptide and PNA5 PLGA polymeric nanoparticles and microparticles.

ATR FTIR molecular fingerprint spectra of pure PNA5 and PNA5-loaded particles are shown in Figure 2. The most typical spectra section for assessing peptide or protein-based drug secondary structure is the amide I area (1710–1590 cm^−1^) [24]. A band at 1634 cm^−1^ in PNA5 spectra can be observed and represents the acidic carbonyl C = O stretching, and the spectra of PLGA MP of both polymers is also observed, however, in PLGA NPs formulations, that band is not present suggesting most of the peptide is entrapped inside the particle and is masked by PLGA bands. The intense band observed at 1762 cm^−1^ is attributed to the stretching vibration of the carbonyl groups present in the two monomers. The bands in the spectra of all formulations show a wide and sharp band of an -OH group, at 3150–3650 cm^−1^ [25].

### 3.3. Differential Scanning Calorimetry (DSC)

**Figure 3:**
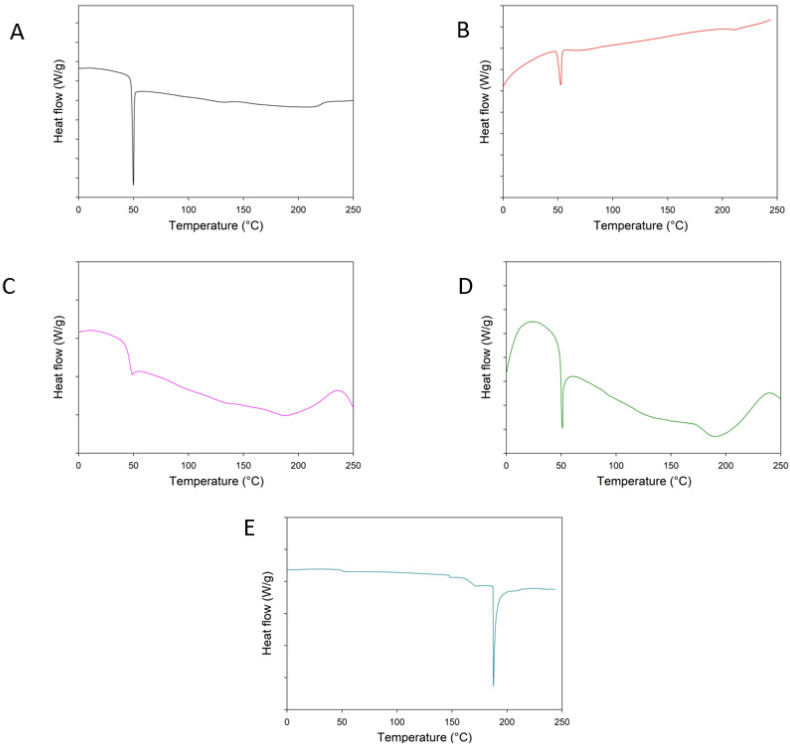
Differential scanning calorimetry (DSC) curves of Raw PNA5 glycopeptide and formulations: (**A**) 503 NPs, (**B**) 503H NPs, (**C**) 503 MPs, (**D**) 503H MPs, (**E**) Raw PNA5.

DSC results of pure PNA5, PLGA NPs, and MPs using both polymers obtained in determining the physical state of the peptide are given in Figure 3. The DSC thermogram of PNA5 (Figure 3D) showed a sharp endothermic peak at 190 °C. On the other hand, two sets of peaks are observed in the DSC thermograms of different formulations as shown in Figure 3; a set of peaks at ~45 °C and an endotherm at ~190 °C. The first peak showed the T_g_ of PLGA, while the second peak set showed the melting point, a first-order phase transition from the solid-state to the liquid state of PNA5.

### 3.4. In Vitro Release of Peptide PLGA Nanoparticles and Microparticles

**Figure 4:**
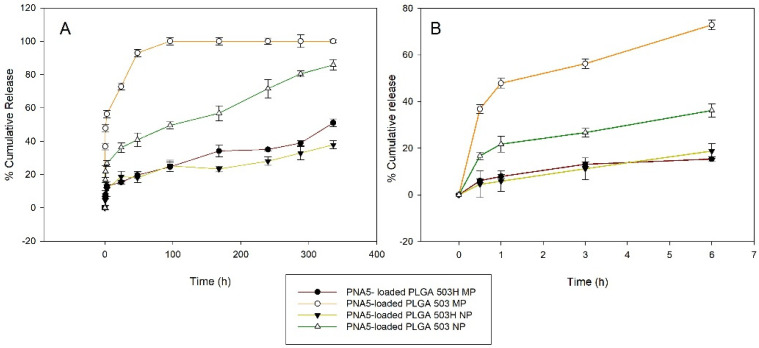
In vitro cumulative release of PNA5 glycopeptide over time from PLGA 503H and PLGA 503 polymeric nanoparticles and microparticles at pH = 7.4 and 37 °C. (**A**) 0–14 d; (**B**) 0–6 h.

In vitro release profiles of PNA5 PLGA NP and MP with different PLGA types are shown in Figure 4. The burst release observed is influenced by its end group composition and size range. Lower burst release was observed for 503H in both NP and MP. For 503 H NPs, after 3 h, % released PNA5 was 12 ± 0.83, in contrast, ester terminated PLGA showed a % release of 56 ± 2.07. Regarding MPs, values of 11.19 ± 4.6 and 26.67 ± 1.8% release of PNA5 were observed for the 503H and 503 MPs, respectively. After burst release, PNA5 was released from PLGA particles in a sustained manner for all formulations, showing release for up to 14 days except for the 503 NPs. At the end of the experiment, for 503H formulation, release values of 51 ± 2.4% and 37 ± 4.5% were observed for PNA5. For ester-capped PLGA, the same trend was observed, NPs release much faster than MP formulation (100 versus 85.78 PNA5 % cumulative release).

## 4. Discussion

The main objective of the study was to prepare and evaluate the release of new MasR agonists PNA5 from PLGA NPs and MPs against cognitive impairment therapy. Previously in our group, PNA5 has shown a poor half-life of 1.0 ± 0.2 h after daily subcutaneous injections [5]. One potential means of addressing this problem is the encapsulation of polypeptide within a PLGA nano/micro particle carrier. These carriers offer the ability to encapsulate a wide variety of molecules, including nucleic acids, proteins and peptides, and small molecules, reducing dosing frequency and the ability of PLGA delivery systems to modify biological compounds’ pharmacokinetics protecting the cargo from hydrolysis and subsequent degradation [22].

Both ester and acid terminated PLGA copolymers successfully synthesized both particle sizes by the w/o/w double emulsification solvent evaporation method. The double emulsion method was used to fabricate the PNA5 peptide-loaded PLGA NPs and MPs as this is the primary method that allows us to entrap highly hydrophilic molecules into a PLGA matrix. An important step in the preparation of particles was the formation of a stable homogenous primary emulsion, and proper energy input in the second emulsion to have the desired final size. The surface charge of particles showed high negative values, which means that the PLGA particles can be stable in suspension (Table 2) due to their strong electrostatic repulsive/attractive interactions. Further, PDI and span values for both sizes showed acceptable values.

The peptide % EE and % DL were highly influenced by the end-capped PLGA (acid or ester-terminated), suggesting that the acid-terminated part of the polymer can interact with any NH_2_ group in the polypeptide chain [26]. Park, Lee [27], showed similar results when encapsulating drug molecules in PLGA; 503H particles showed a higher EE and DL than its ester-capped PLGA. The values of % DL in this article are comparable with commercial Lupron Depot^®^, which uses PLGA MPs and a Leuprolide acetate peptide with a DL of 8.5% [28]. It should be noted that only when the 503H is used for PLGA NP/MPs that this value can be achieved. Furthermore, the ionic interactions of peptides binding to polyesters can be explained by the observed behavior. Positively charged peptide moieties and negatively charged COOH end groups have been shown to interact via ionic forces, enhancing drug encapsulation and initial burst release [29]. This result could explain why acid carboxyl end-capped PLGA has a higher percentage of % EE and % DL than ester end-capped PLGA. More peptide molecules are attracted to polymers with more significant acid numbers because they have more negatively charged moieties.

DSC thermograms show the PNA5 endothermic peak as consistent with the literature [14]. The glass transition temperature of PLGA is responsible for the endothermic peak exhibited in all PLGA thermograms at about 48–50 °C [30]. The sharp endothermic PNA5 peak disappeared from the DSC curve of the PNA5-loaded PLGA particles formulation. The complete disappearance of the active substance peak could be attributed to either the formation of a homogeneous polymeric matrix or the dilution effect of the polymer [31]. The disappearance of the endothermic PNA5 peak in DSC curves of prepared particles indicated peptide incorporation, homogenous matrix formation, and amorphous structure [32].

Finally, different behavior was observed in the in vitro release study. A faster release of the polypeptide from nanoparticles was expected since the hydrolysis of the polymer is accelerated due to faster water penetration and acid-catalyzed degradation of the polyester matrix. Brauner, Schwarz [15], synthesized both PLGA NP and MP for trimethoprim for instillative treatments of urinary tract infections. They showed similar results to our study, where with NPs formulation of both polymers, the dissolution rate is higher than MPs. In their study, after 10 h of trimethoprim release, nearly 100% was completely released from the NP system and MPs formulation was around 75% using 503H polymer. Drug release from polymeric systems during the first phase, the active agent is released primarily through its diffusion through the polymer matrix, whereas during the second phase, the release is mediated by both drug diffusion and polymer matrix degradation. During polymer hydration, drug diffusion through the polymer matrix occurs slower [33]. To evaluate the drug release process and investigate the role of polymer in the dissolution profile, mathematical models were primarily used to predict the release of the encapsulated PNA5 as a function of time. As a result, the mathematical evaluation of our formulation’s drug release kinetics was fitted against zero order, first order, Higuchi and Korsmeyer, and Peppas models (Table 3). This will ensure optimal pharmaceutical formulation(s) design as well as a better understanding of the release mechanism(s) through experimental verification [34].

Both PLGA 503H and PLGA 503 nano/microparticles correlated well to the Higuchi model (adjusted R^2^ 0.958 and 0.956 for NPs; 0.921 and 0.98, respectively) and Korsmeyer-Peppas (adjusted R^2^ 0.964 and 0.912 for NPs; 0.984 and 0.973, respectively) models. The diffusion exponent *n* of the Korsmeyer-Peppas model was between 0.17 and 0.35 suggesting a Fickian diffusion. The *n* value indicates the mechanisms to describe how the active compound is released from its matrix. In this case, the solvent diffusion is much greater than the process of polymeric chain relaxation. The kinetics of this phenomenon are characterized by diffusivity [35]. If *n* is less than 0.43 in the case of spherical encapsulation shape, then a Fickian diffusion release mechanism is implied [36]. The zero-order model describes drug release from a system in which the content is released at a constant rate independent of concentration. The first-order model describes a system in which the residual drug concentration uniquely determines the drug release rate. In addition, the Higuchi model hypothesizes that the system’s edge effect must be negligible for drug diffusion and the dissolution and swelling of the system are irrelevant, showing a constant drug diffusivity. Korsmeyer-Peppas is another complete semi-empirical model that can be used to generalize the various release phenomena involving either diffusion or swelling [37,38].

Particles in the nanosized range have a greater surface area to volume ratio than particles in the micronized range, resulting in a faster dissolving rate, aided further by Brownian motion. This increased surface area of the nanoparticle should ideally allow for greater water contact/penetration into the particles while also allowing for faster degradation [39]. Due to the much faster drug release of 503 formulations, the nanometer and micrometer particles synthesized with the acid-capped PLGA seem to be better for PNA5 administration and improve its cognitive impairment effect in patients. Encapsulation peptides in PLGA diblock copolymer for sustained release injectable drug delivery continues to have significant clinical significance, as evidenced by multiple marketed pharmaceutical peptide PLGA products used clinically (e.g., leuprolide PLGA and octreotide PLGA) as sustained-release injectables [18] and continued research in this exciting area of peptide drug delivery [21,40,41].

## 5. Conclusions

This comprehensive and systematic study reports for the first time on the successful design and development of PNA5 loaded in the FDA-approved biocompatible biodegradable diblock copolymer PLGA as NPs and MPs by double emulsion solvent evaporation technique, comprehensive physicochemical characterization, and in vitro drug release. The formulations showed an acceptable PDI and span value for PLGA particles prepared by double emulsion, indicating monodisperse particles. The release of PNA5 from the particles was first with burst effect for both size and polymer used. The release was then slower and prolonged for up to 2 weeks for some formulations. The DSC thermal analysis and ATR FTIR molecular fingerprinting spectra analysis of the PNA5 PLGA particles revealed favorable intermolecular interaction between PNA5 and PLGA.

In this study, PLGA with the same molecular weight but different end-capping (acid or ester terminated) was used to compare the effects in formulation characterization. These end groups had an impact on properties, particularly the EE percent and release rate. The size of the particle also affected the release behavior due to the different surface-area ratios. The NP prepared with the polymer having the ester end-group released faster, whereas the NP prepared with only the acid end group released up to 14 days with 40% of PNA5 released. In terms of polymer selection, PLGA R503H provided several advantages over PLGA R503 in this study including higher drug loading, combined with a more sustained release over time, resulting in less burst release at the first sampling point.

Because the study of drug release kinetics provides critical information for better realization and optimization of nanoparticulate drug delivery, various methodologies for determining release kinetics from such formulations have been developed. The Higuchi model was the best-fitting mathematical model (among zero and first-order methods) for showing drug release patterns from PLGA particles in this study. The *n* values of Korsmeyer-Peppas are mostly less than 0.5, suggesting the release mechanism was governed by diffusion in all formulations. Regardless of the PLGA type used, both nanoparticles and microparticles demonstrated innovative PNA5 sustained-release formulations.

## Figures and Tables

**Table 1:** PNA5 loaded-PLGA microparticles (MPs) and nanoparticles (NPs): composition of formulations and manufacturing parameters.

Formulation	PLGA (mg)	Oil Phase (mL)	PNA5 (mg)	W_1_ Phase (mL)	W_2_ Phase (mL)	W_3_ Phase (mL)	E_1_ (min)	E_2_ (min)
NPs	20	1	2	0.2	8	-	1	1
MPs	50	5	10	0.5	20	50	5	1

**Table 2:** Characteristics of nanoparticles (NPs) and microparticles (MPs) prepared from both PLGA types. EE = encapsulation efficiency; DL = drug loading; ζ = zeta potential; DI = polydispersity index. (*n* = 3).

Formulation		% EE	% DL	ζ Potential	Size (µm)	PDI	Span
NPs	503	10.8 ± 0.9	3.4 ± 0.2	−25 ± 1.5	0.769 ± 0.089	0.27 ± 0.04	-
	503H	42 ± 1.5	8.3 ± 0.4	−30.4 ± 1.9	0.35 ± 0.007	0.34 ± 0.02	-
MPs	503	13 ± 0.4	5.4 ± 0.5	−24 ± 2.3	3.7 ± 0.7	-	0.93 ± 0.01
	503H	55 ± 2.3	9.3 ± 0.5	−32 ± 2.5	2.4 ± 0.3	-	0.63 ± 0.04

**Table 3:** Mathematical models and kinetic constants.

Model	503H NP			503 NP			503H MP			503 MP		
	K (h^−1^)	R^2^	*n*	K (h^−1^)	R^2^	*n*	K (h^−1^)	R^2^	*n*	K (h^−1^)	R^2^	*n*
Zero-order	0.11	0.90	-	0.19	0.45	-	0.08	0.80	-	0.20	0.88	-
First-order	0.002	0.49	-	0.002	0.12	-	0.002	0.44	-	0.003	0.33	-
Higuchi	2.12	0.95	-	7.88	0.95	-	1.57	0.92	-	3.57	0.98	-
Korsmeyer and Peppas	5.40	0.96	0.35	6.66	0.91	0.27	20.2	0.98	0.19	44.8	0.97	0.17

## Data Availability

The data presented in this study are available on request from the corresponding author.
